# Effect of different ceramic materials and dentin sealing on occlusal veneers bond strength and fracture resistance

**DOI:** 10.1186/s12903-025-05505-5

**Published:** 2025-02-05

**Authors:** Dina Mohamed Nasr, Rewaa Gaber AboElHassan

**Affiliations:** 1 Operative Dentistry, Conservative Dentistry Department, Faculty of Dentistry, Alexandria University, Alexandria, Egypt; 2 Fixed Prosthodontics, Conservative Dentistry Department, Faculty of Dentistry, Alexandria University, Alexandria, Egypt

**Keywords:** Occlusal veneers, CAD/CAM, Immediate dentin sealing, Delayed dentin sealing, Debonding forces, Bond strength, Fracture resistance, Fractographic analysis

## Abstract

**Background:**

The effect of immediate dentin sealing (IDS) or delayed dentin sealing (DDS) on bond strength of different ceramic occlusal veneer restorations, was not examined yet, but the digital quantification of surface area of preparations helped in the calculation of the actual bond strength of these restorations, besides that, dentin sealing could influence the fracture resistance values of different ceramic occlusal veneer restorations.

*This study aimed to* examine the effect of different dentin sealing strategies and different restorative materials on the debonding forces, bond strength and fracture resistance of occlusal veneer restorations.

**Materials and methods:**

Human mandibular molars (*N* = 64/test) were prepared for occlusal veneer restorations fabricated from Lithium disilicate ceramic (LS) (control group), Zirconia-reinforced lithium Silicate ceramic (ZLS), High translucent zirconia ceramic (HZ), and Resin nano ceramic (LU) (*n* = 16). Each material was subdivided according to IDS or DDS application, (*n* = 8). Bond strength testing and fracture resistance values were measured using universal testing machine. Two-way ANOVA was performed to investigate the effects of the two variables, dentin sealing and material selection, and their interaction (α = 0.05), followed by One-way ANOVA and post hoc or Student t-test for further determination of significance.

**Results:**

For debonding forces and bond strength testing there were significant differences regarding different materials and dentin sealing strategies, while for fracture resistance, the material selection had a significant effect on it with no significant effects of dentin sealing strategies. The interaction between materials and dentin sealing showed no significant differences concerning all tests. For debonding forces and bond strength there was a higher result related to IDS application than DDS (*p* < 0.001*), and there was a significant effect of material selection on them (LS > ZLS > HZ > LU), (*p* = 0.001^*^). Regarding the fracture resistance there was significant increase of all studied materials than LU (*p* = 0.002^*^).

**Conclusion:**

Immediate dentin sealing increased the required debonding forces of occlusal veneers made of different materials and their bond strength without significant effect on their fracture resistance, however this improvement was found to be material dependant. All the used materials can withstand lateral and occlusal forces higher than the values recommended for restoring posterior teeth.

## Introduction

Tooth wear is the main cause for loss of vertical dimension of occlusion, its progression can lead to exposure of dentin with a resultant dentin sensitivity [1]. The continuous advancement of adhesives, with the introduction of adhesive restorations, such as the occlusal veneers, led to restoration of moderate to severe tooth wear with minimal preparation [2].

When restoring moderate to severe tooth wear, it is recommended to increase the vertical dimension of occlusion by 2–5 mm, so occlusal veneers commonly had (1.5–2.5 mm) thickness [3]. The development of computer-aided design and computer-aided manufacturing (CAD/CAM) technology, advanced ceramics, appropriate bonding materials, have enabled the manufacturing and application of satisfactory dental restorations [4, 5].

Most dental ceramic materials consist of an amorphous part SiO_2_ (glass), and crystals. The amount and size of crystals adjust the mechanical properties of dental ceramics while the amorphous part provides their translucency and natural looking, besides that it guarantees good chemical bond with resin cements [6]. However, the relatively low fracture toughness of glass ceramics could be a major restriction for their use in different prosthodontic solutions Silva et al., 2017 [7].

Consequently, the use of polycrystalline ceramics (zirconium dioxide) offered excellent mechanical properties, with higher fracture toughness and excellent wear behavior but with lower translucency due to absence of glass phase which also make it resistant to etching with hydrofluoric acid [6, 8]. The introduction of monolithic high translucent zirconia is based on adding higher ytteria content for fully stabilization of cubic phase with larger grain size, together with increasing the sintering time and temperature for enhanced crystallization and less porosities, in addition to lowering alumina content and impurities, all of these factors are responsible for added translucency and better esthetics, so they could be a good solution for esthetic posterior restorations to endure chewing forces and generated mechanical stresses at thin thicknesses [7–9].

There is a debate about the most suitable type of CAD/CAM materials that can be used for indirect occlusal veneers restorations. Lithium disilicate had been considered the most common and strongest glass ceramic material and it was upgraded by the addition of zirconia particles for improvement of flexural strength of lithium silicate structure [4, 8]. Nevertheless, the improvement of flexural strength does not certainly mean high damage tolerance as occlusal veneers made of a CAD/CAM composite with lower flexural strength and higher elasticity exhibited higher fracture resistance under cyclic fatigue testing compared to two CAD/CAM glass ceramics, Schlichting et al., and Magne et al., [10, 11].

On the other hand, CAD/CAM hybrid ceramics as Resin nano ceramic materials have been considered as adequate replacement to glass–ceramics because they can withstand high masticatory forces and dynamic fatigue in thin thicknesses [12, 13].

Amongst the previously tested materials in literature Zamzam et al., 2021 [4], found that zirconia occlusal veneers presented the highest resistance to failure under lateral loading and debonding was the mostly presented failure type, in comparison to fractures at lower loads for hybrid ceramics and lithium disilicate, whereas, Ioannidis et al., (2019 and 2020) found that all tested minimally invasive occlusal veneers made of different dental biomaterials exhibited considerably higher fracture resistance than normal occlusal forces with clinically irrelevant statistically significant differences between different materials [14, 15].

Tooth preparation for indirect ceramic bonded restorations could lead to dentin exposures, thus it was recommended to seal the freshly cut dentinal tubules with a dental adhesive immediately after tooth preparation following the immediate dentin sealing (IDS) protocols, as recommended by Pascal Magne (2005) [16], this could achieve higher bond strength, lesser gaps, diminished bacterial leakage, and reduced dentin sensitivity which would aid in restoring teeth for patients suffering from worn dentition [17–20].

The availability of CAD/CAM technology in the clinic directed the clinicians to use the IDS directly after the preparation followed by direct cementation of restoration without any temporization period, while the other technique is to use IDS directly after the preparation and cementation of the final restoration after a temporization period, which is yet considered the most common in dental practice due to unavailability of CAD/CAM units in most of dental clinics [21]. On the contrary, in Delayed Dentin Sealing (DDS), the dentin adhesive is applied just before cementing the restoration after a temporization period which could have a negative effect on bond strength of restorations [21, 22].

There is a lack of data regarding the bond strength of occlusal veneer restorations made from different ceramic materials and bonded to the tooth structure, by using different dentin sealing protocols but the ability to measure the surface area of prepared tooth surface would help its quantification through the use of 3D CAD software programs where the surface topography of the prepared teeth is measured by counting the number of triangulation points on the 3-D surface of every abutment tooth at each STL (**S**tandard **T**essellation **L**anguage) file [23]. Besides that, dentin sealing could influence the fracture resistance values of different ceramic occlusal veneer restorations [24]. Also, it is important to predict the sustainable success of all-ceramic restorations after suffering from stresses occurring from the physicochemical alterations of restorative materials during thermocycling, and from those occurring by occlusal stresses [25].

The aim of this study was to determine the effect of immediate and delayed dentin sealing on the bond strength and fracture resistance of CAD/CAM occlusal veneers fabricated from lithium disilicate ceramic, Zirconia reinforced lithium silicate ceramic, High translucent zirconia ceramic and resin nano ceramic for restoration of mandibular molars and to determine their failure in bonding and their fracture modes.

The null hypotheses of this study stated that there were no significant differences between immediate dentin sealing or delayed dentin sealing using different restorative materials on the debonding forces and the resultant bond strength nor on the fracture resistance of occlusal veneer restorations.

## Material and methods

The minimal sample size was calculated based on a previous study that aimed to compare the effect of dentin sealing and different bonding materials on shear bond strength of reinforced glass- ceramic overlays [26], adopting a power of 80% (b = 0.20) to detect a standardized effect size in resistance to forces (primary outcome) of 0.847, and level of significance 5% (α error accepted = 0.05), the minimum required sample size was found to be 6 teeth per group and it was increased to 8 teeth /subgroup to overcome the processing errors (number of groups = 8) (Total sample size = 64 teeth/ test) [27]. The sample size was calculated using G*Power version 3.1.9.2 [28].

One hundred twenty-eight freshly extracted intact human mandibular second molars, were collected. Teeth extracted due to periodontal reasons, without any caries, fillings, or severe attrition. The study was performed in accordance with ethical guidelines of Declaration of Helsiniki. To standardize the teeth’s size, a digital caliper (Titan Electronic Digital Caliper, Pennsylvania, USA) was used to measure the mesiodistal and bucco-lingual dimensions, and 0.5 mm difference was the maximum variation that could be accepted [24].

### Teeth selection and disinfection

All selected teeth were cleaned and kept in a 0.1% thymol solution for 4 weeks [4]. Each tooth was embedded in a self-curing acrylic resin 2 mm below the cemento- enamel junction using a split metallic copper mold, half of the teeth were randomly assigned for the fracture resistance test and to simulate the periodontal ligament, the roots of all teeth were dipped into melted utility wax before embedding into the acrylic resin to produce nearly 0.2 to 0.3 mm layer then it was replaced by Polyvinyl siloxane material (PERFIT light body-Normal Set A-SILICON, HUGE DENT, China) [4, 29].

### Teeth preparation

**Fig. 1:**
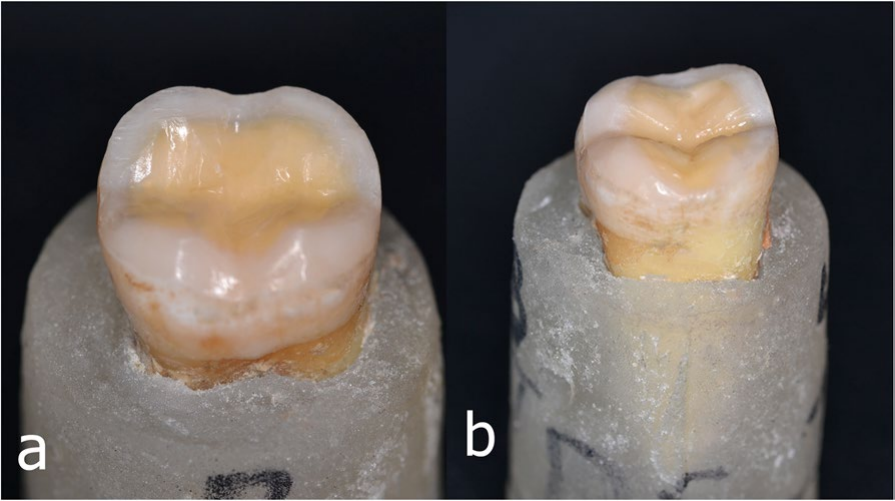
Prepared mandibular molar to receive occlusal veneere restoration, (**a**) buccal view, (**b**) proximal view

A pre-preparation silicon index was fabricated for each tooth using additional silicone impression material (PERFIT Putty-Normal Set A-SILICON, HUGE DENT, China). The tooth preparation was done using high-speed diamond rotary instruments (TR-12, DIA-BURS, MANI, Ut- sunomiya, Japan) and the diamond burs were changed every five preparations [24]. All preparations were performed by a single operator. For all teeth, the occlusal surface was reduced by 1.5 mm at the cusp tip and central groove to expose dentin, following the occlusal anatomy, without any steep inclinations of cusps and all sharp angles were rounded [2, 30]. The peripheral enamel on the buccal axial wall were prepared by an inclined plane using only the tip of the bur, this was done for more favourable adhesion through cutting enamel prisms almost perpendicular to their longitudinal axis and for maximum preservation of sound residual tissue [31]. The occlusal clearance was checked by the prepared silicon index by the aid of a periodontal probe and all line angles and margins were rounded and finished using Dura- White Stones (CN1 024–0201, SHOFU Dental, Kyoto, Japan) to create a perfectly smooth surface [31], (Fig. 1a, b).

The prepared teeth for bond strength and fracture resistance tests (*N* = 64/test) were divided according to different ceramic restorative materials for the fabrication of occlusal veneer restorations into four material groups: Lithium disilicate ceramic occlusal veneers, they were milled from IPS e- max CAD (LS) (Ivoclar Vivadent AG, Schaan, Liechtenstein) (control group) (*n* = 16/test), Zirconia reinforced lithium silicate ceramic occlusal veneers, they were milled from Vita Suprinity (ZLS) (Ivoclar Vivadent AG, Schaan, Liechtenstein), (*n *= 16/test), High translucent zirconia ceramic occlusal veneers, (HZ) they were milled from Prettau® Zirconia (Zirkonzahn, Gais, Italy), (*n* = 16/test), and Resin nano-ceramics occlusal veneers, they were milled from Lava Ultimate (LU) (3 M ESPE, Saint Paul, Minnesota, United States) (*n* = 16/test).

Each material group was further subdivided into two subgroups according to the used technique of dentin sealing (IDS and DDS):

- *1- Immediate dentin sealing (IDS);* the exposed dentin surface was sealed directly after the preparation using a layer of universal bonding agent (All Bond Universal; Bisco, Schaumburg, IL, USA), air thinned and cured for 10 s, for prevention of provisional restoration adhesion to the sealed dentin surface, it is recommended to apply glycerine gel onto the cured adhesive with subsequent 10 s light curing through it for prevention of oxygen inhibition layer formation [26], followed by refreshment of surrounding enamel margins by the tip of diamond bur to remove any excess of cured adhesive resin [11]. The teeth were cleaned and scanned by an intraoral scanner CS 3700 (Carestream CS3700, Carestream Dental LLC, Atlanta, GA, United States) followed by placement of 2 mm thickness provisional restoration (Perfectemp II, DenMat Holdings LLC, Central Ave, Lompoc, United States) using a circumferential tofflemire band securely adjusted around the teeth, then stored in artificial saliva at 37 °C for two weeks as a temporization period [21].
- *2- Delayed dentin sealing (DDS);* the exposed dentin surface was not sealed by adhesive after preparation, and the teeth were scanned by an intraoral scanner CS 3700 followed by placement of 2 mm of provisional restoration material (Perfectemp II) following the previous technique, after that they were stored in artificial saliva at 37 °C for two weeks temporization period [21].

**Fig. 2:**
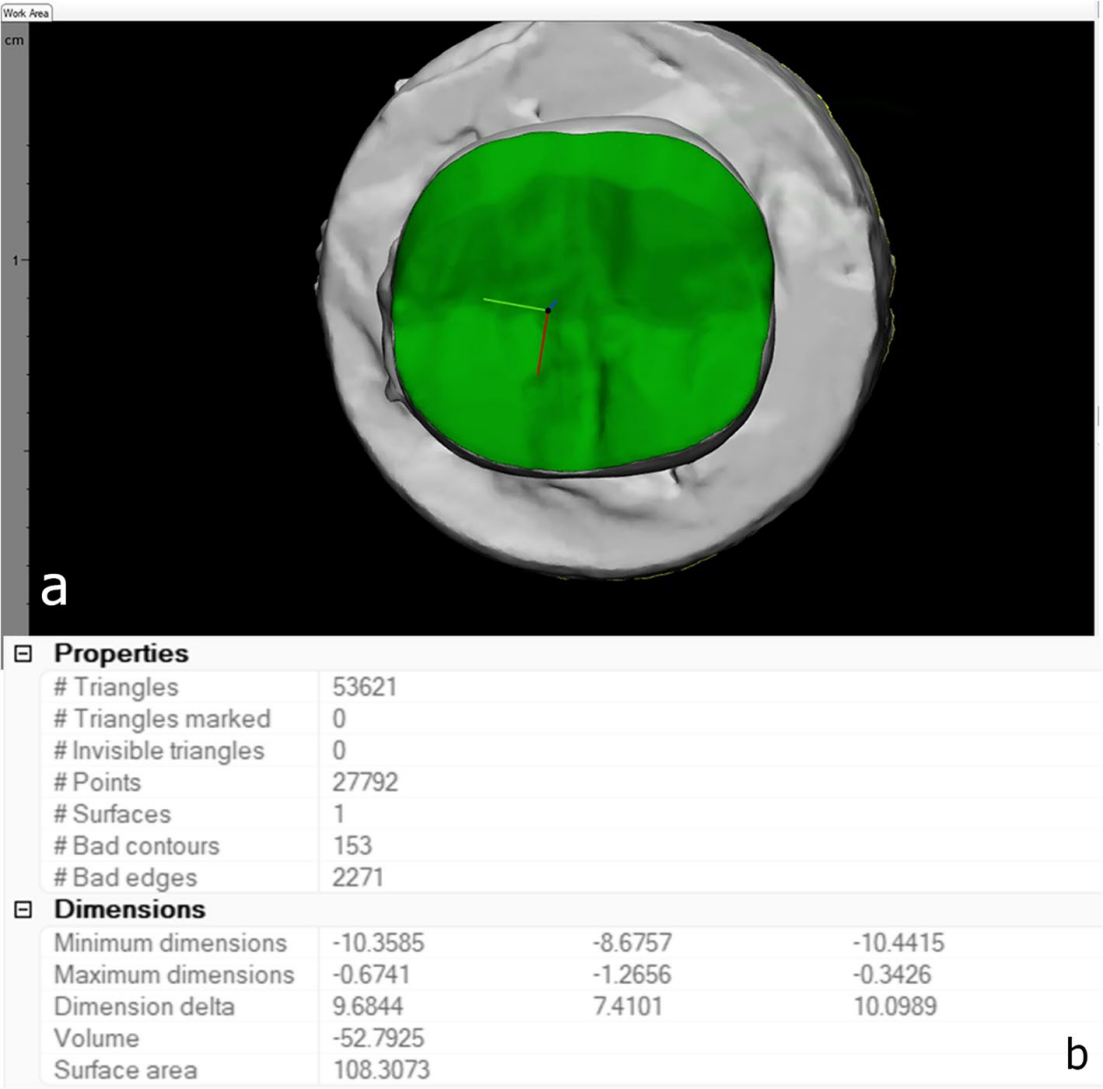
Calculation of surface area of prepared teeth, (**a**) Marking of prepared surface; (**b**) The properties’ menu showing details of surface area calculations

The scanned teeth after preparation were saved to a CAD software program (Exocad, Germany), in the form of STL files which were imported into 3-matic software (Mimics innovation suite, Materialize, Belgium). The prepared surface was marked using wave brush mark tool. Afterwards, the marked surface was selected, and the surface area was obtained from the properties’ menu. Surface area was automatically determined in mm^2^ by the software through counting the number of triangulation points and triangles on the 3D surface of prepared occlusal surface of every abutment tooth [23, 26], (Fig. 2a, b).

### Occlusal veneers fabrication

Occlusal veneer restorations were designed with a consistent thickness of (1.5 mm), with 50 µm cement space. The designed occlusal veneers were sent for milling. The preparations’ designs were randomly allocated to the four studied materials’ groups and subgrouping according to dentin sealing technique for (LS + IDS and LS + DDS), (ZLS + IDS and ZLS + DDS), (HZ + IDS and HZ + DDS) and (LU + IDS and LU + DDS). All milled occlusal veneers were finished and glazed according to the manufacturers’ instructions for each. After two weeks (temporization period), the provisional restorations were removed with an ultrasonic tip and a scaler, all surfaces of the prepared teeth were cleansed gently with a soft brush and fluoride-free pumice [16]. The milled restorations were checked regarding their adaptation, fitness to their respectively prepared teeth, and they were checked regarding their thicknesses.

### Bonding procedures

Following the selective enamel etching technique, 37% phosphoric acid etching gel (BISCO, USA) was applied only on the enamel for 15 s, then rinsed thoroughly with a stream of water for at least 15 s and dried with compressed air just for cleaning [32, 33], followed by a new adhesive layer agent (All Bond Universal; Bisco, Schaumburg, IL, USA) applied on the tooth surface (enamel and dentin) and left without curing to prevent any interferences with the complete seating of the restorations if it is cured [16].

Restorations were cleaned with ultrasonic and degreased with ethanol, for the cementation of (LS, and ZLS restorations) the intaglio surfaces were treated with hydrofluoric acid (Porcelain Etchant, Bisco, Schaumburg, IL, USA) for 20 s for LS and 30 s for ZLS [34], then, silane (porcelain primer, Bisco, Schaumburg, IL, USA) was applied and left to work for 60 s and dried for 5 s with oil-free air. For (HZ, and LU restorations) the intaglio surfaces were abraded using 50 µm Aluminium oxide particles (AL_2_O_3_) (Renfert, Hilzingen, Germany) at a pressure of 2 bars from 10 mm distance and perpendicular to the surface, then the zirconia primer Z-Primer Plus (Bisco, Schaumburg, IL, USA) was applied onto the intaglio surface of HZ restorations while silane (porcelain primer) was applied onto the LU restorations according to the manufacturer’s recommendations for each [4].

Finally, Duo-Link Universal (dual-cured resin luting cement; Bisco, Schaumburg, IL, USA) was applied onto the restoration and the preparations. Each occlusal veneer was first seated onto its respective prepared tooth gently. Then, a dead weight of 1 kg was applied by using a customized loading apparatus on the occlusal surface of the veneer. After 2 s of tack curing with a LED curing light (Bluephase 20i LED Curing Light, wavelength 385—515 nm, Output 2,000 mW/cm2, Ivoclar Vivadent AG; Schaan, Liechtenstein), excess cement was removed. Each surface of the tooth was exposed to light curing for 20 s for each quarter surface (mesio-facial, disto-facial, disto-lingual, mesio-lingual). The margins were inspected, and any remaining excess cement was removed by scalpel [4, 35]. The margins were finished and polished with diamond ceramic polishers (Identoflex™ Diamond Ceramic Polishers, Kerr, California, USA) for all groups, then each specimen was stored in distilled water at ambient temperature for 24 h before testing [10]. The bonded restorations were subjected to thermocycling in distilled water for 10,000 cycles by a thermocycling apparatus, between 5 and 55ºC, the dwell time at each temperature was 30 s and the transfer time was 2 s which was clinically equivalent to approximately one year of clinical service [36].

### Bond strength test

**Fig. 3:**
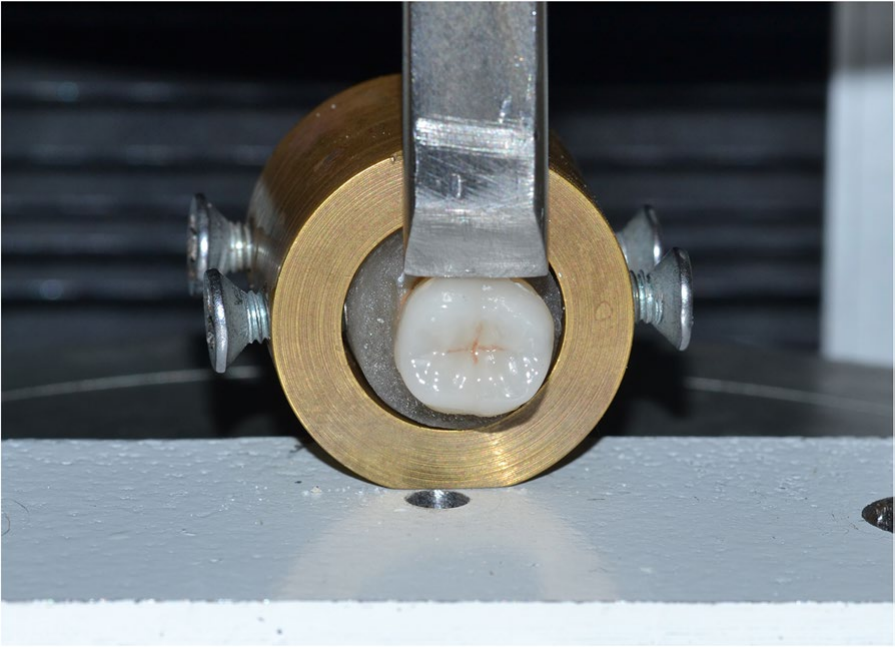
Application of bond strength test

Each acrylic block containing the teeth with the bonded occlusal veneers from the four studied groups, were fixed to the metallic holding attachment at 90° to the vertical plane of the universal testing machine (5ST, Tinius Olsen, England). The ceramic restorations were subjected to debonding forces applied within 1 mm from the tooth/veneer interface using a stainless-steel blunt knife edge probe for creation of debonding forces at cross head speed of 0.5 mm/min until bonding failed, (Fig. 3). The debonding force (N) was recorded automatically at the point of failure, and bond strength (MPa) = Load (N)/area (mm^2^)], where the surface area was pre-measured during designing of restorations step [26]. The three parameters of this test (surface area, debonding forces, and bond strength) were statistically analysed.

After debonding, the de-bonded interfaces of the specimens were examined to determine the failure modes as follows: (1) adhesive failure (adhesion failure between resin cement and dentin or between cement and restorations); (2) cohesive failure (failure in restorative materials or the bonded substrate); (3) mixed failure (partial adhesive failure between dentin and cement, and partial between cement and restorations), [37].

### Fracture resistance test

**Fig. 4:**
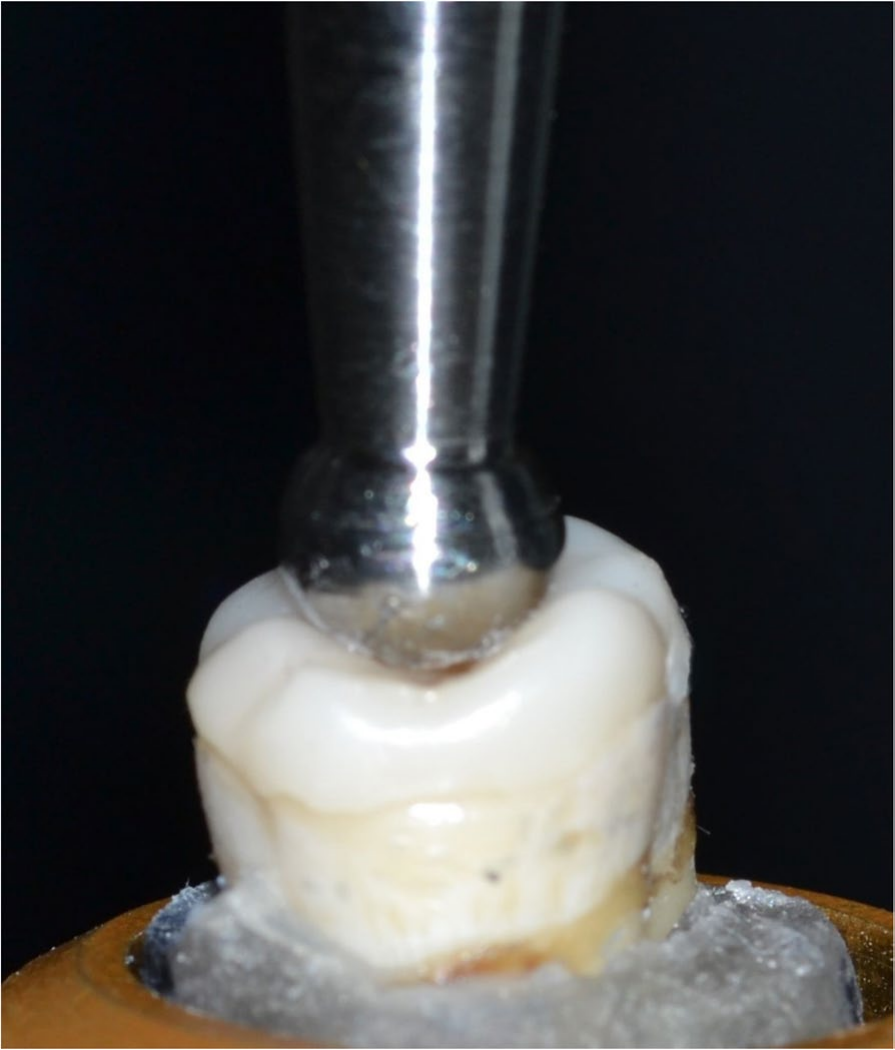
Application of fracture resistance test

A 6-mm-diameter metal sphere attached to a 5 kN load cell was positioned over the central fossa in the occlusal surface of the occlusal veneer to achieve tripoding of contact points along the inclination of cusps, using the universal testing machine where the compressive loads were applied at a crosshead speed of 1.0 mm/min, (Fig. 4). The maximal values of force were documented in Newtons (N) [24].

The restored teeth after fractured were visually examined using *digital single-lens reflex camera* (DSLR) Nikon d5300 ( NIKON, Tokyo, Japan) accompanied with Sigma 105 mm f/2.8 EX DG OS macro lens (Sigma, Kanagawa, Japan) and they were classified as follows a) reparable fracture that occurred in the occlusal veneer only, or fracture that involved one or more cusps); or b) irreparable fractures (the restored tooth was longitudinally divided into two parts or more at the level of the pulp chamber floor) [24, 38].

### Fractographic analysis

Representative fractured specimens were mounted on metallic stubs, gold sputter-coated by (JEOL Fine Coat Ion-sputter JFC-1100, JEOL, Tokyo, Japan) for fractographic analysis by scanning electron microscopy (JEOL JSM-5510 LV InTouchScope, Tokyo, Japan) at 35 × magnification.

### Statistical analysis

Data were fed to the computer and analysed using IBM SPSS software package version 20.0. (Armonk, NY: IBM). Data were tested for normality by the Shapiro–Wilk test. For normally distributed quantitative variables Two-way ANOVA test was used to show the effect of materials and dentin sealing on Debonding forces, Bond strength and Fracture resistance, followed by One-way ANOVA and Post Hoc test (Tukey) for comparing the different studied groups, or by using Student t-test. Significance of the obtained results was judged at the 5% level.

## Results

For adequate standardization of the work done besides measurement of teeth during their selection, the measured surface areas of all submitted teeth to bond strength test were statistically analysed and there was no significant difference between the teeth in different groups (*p* = 0.067), where the surface area ranged from (95.7834 to 111.3634 mm^2^) with low ratio of coefficient of variation between groups.

Two-way ANOVA test of debonding forces and bond strength found significant differences between different materials and between dentin sealing strategies, while for fracture resistance the material selection had a significant effect on it with no significant effects of dentin sealing, the interaction between the 2 variables (materials and dentin sealing) showed no significant differences regarding the debonding forces, bond strength and fracture resistance as presented in (Table 1).

**Table 1:** Two-way ANOVA used to show the effect of materials and dentin sealing on Debonding forces (N), Bond strength (MPa) and Fracture resistance (N)

	**Source**	**Type III Sum of squares**	**df**	**Mean Square**	**F**	***p*-value**
**Debonding forces**	**Materials**	418701.254	3	139,567.085	10.004	< 0.001^*^
	**Treatment**	308912.768	1	308,912.768	22.144	< 0.001^*^
	**Materials × Treatment**	107622.964	3	35,874.321	2.572	0.063
**Bond strength**	**Materials**	34.042	3	11.347	9.017	< 0.001^*^
	**Treatment**	28.223	1	28.223	22.428	< 0.001^*^
	**Materials × Treatment**	8.423	3	2.808	2.231	0.095
**Fracture resistance**	**Materials**	1262063.771	3	420,687.924	5.694	0.002^*^
	**Treatment**	144817.351	1	144,817.351	1.960	0.167
	**Materials × Treatment**	184770.654	3	61,590.218	0.834	0.481

^*^Statistically significant at *p* ≤ 0.05

For debonding forces and bond strength, the main effect of materials was investigated after eliminating the dentin sealing factor. The results are presented in (Tables 2 and 3 respectively), for debonding forces there was significant increase in the bond strength for all materials than LU (*p* < 0.001^*^), LS showed the highest force (644.5 ± 149.9 N), followed by ZLS (585.7 ± 140.2 N) and HZ (559.3 ± 154.9N) while the lowest was LU (423.8 ± 117.1N). Regarding the bond strength there was significant increase in the bond strength for LS and ZLS than LU, but for HZ there was no significant difference with the other studied materials (*p* = 0.001^*^), the highest bond strength was obtained by LS (6.12 ± 1.48 MPa), followed by ZLS (5.55 ± 1.27 MPa) and HZ (5.37 ± 1.43 MPa), while the lowest was LU (4.12 ± 1.13 MPa).

**Table 2:** Comparison between the studied different materials according to Debonding forces (N)

Debonding forces	Material				F (p)
	**LS** **(*n* = 16)**	**ZLS** **(*n* = 16)**	**HZ** **(*n* = 16)**	**LU** **(*n* = 16)**	
Min.	359.1	391.0	370.5	253.3	*F* = 6.991^*^ (*p* < 0.001^*^)
Max.	872.0	856.5	862.9	600.3	
Mean	644.5^a^	585.7^a^	559.3^a^	423.8^b^	
± SD.	149.9	140.2	154.9	117.1	
Median	666.3	562.1	512.0	432.9	

*SD* Standard deviation, *F* One way ANOVA test, Pairwise comparison between each 2 groups was done using Post Hoc Test (Tukey)

^*^Statistically significant at *p* ≤ 0.05. Means with totally Different letters ^(a−b)^ are significant

**Table 3:** Comparison between the studied different materials according to bond strength (MPa)

Bond strength (MPa)	Material				F (p)
	**LS** **(*n* = 16)**	**ZLS** **(*n* = 16)**	**HZ** **(*n* = 16)**	**LU** **(*n* = 16)**	
Min.	3.48	3.67	3.49	2.48	*F* = 6.356^*^ (*p* = 0.001^*^)
Max.	8.45	7.90	8.61	5.76	
Mean	6.12^a^	5.55^a^	5.37^ab^	4.12^b^	
± SD.	1.48	1.27	1.43	1.13	
Median	6.16	5.33	5.03	4.40	

*SD* Standard deviation, *F* One way ANOVA test, Pairwise comparison between each 2 groups was done using Post Hoc Test (Tukey), *p p* value for comparing between the studied different subgroups

^*^Statistically significant at *p* ≤ 0.05. Means with totally Different letters ^(a−b)^ are significant

Concerning the effect of dentin sealing on debonding forces and bond strength with eliminating the effect of material selection, there was an overall significant increase with the use of immediate dentin sealing (IDS) than delayed dentin sealing (DDS), (*p* < 0.001^*^), as presented in (Tables 4 and 5 respectively). IDS showed higher forces and bond strength (622.8 ± 169.9 N, 5.96 ± 1.59 MPa respectively) than DDS (483.9 ± 115.3 N, 4.63 ± 1.06 MPa respectively).

**Table 4:** Comparison between the two studied different treatments according to Debonding forces (N)

Debonding forces (N)	Treatment		t	*p*
	**IDS** (*n* = 32)	**DDS** (*n* = 32)		
Min.	253.3	261.3	3.827^*^	< 0.001^*^
Max.	872.0	728.2		
Mean	622.8	483.9		
± SD.	169.9	115.3		
Median	635.1	475.8		

*SD* Standard deviation, *t* Student t-test, *p* *p* value for comparing between the two studied treatments

^*^Statistically significant at *p* ≤ 0.05

**Table 5:** Comparison between the two studied different treatments according to bond strength (MPa)

Bond strength (MPa)	Dentin sealing		t	*p*
	**IDS** **(*n* = 32)**	**DDS** **(*n* = 32)**		
Min.	2.49	2.48	3.936^*^	< 0.001^*^
Max.	8.61	6.98		
Mean	5.96	4.63		
± SD.	1.59	1.06		
Median	5.96	4.67		

*SD* Standard deviation, *t* Student t-test, *p p* value for comparing between the two studied Dentin sealing strategies

^*^Statistically significant at *p* ≤ 0.05

**Fig. 5:**
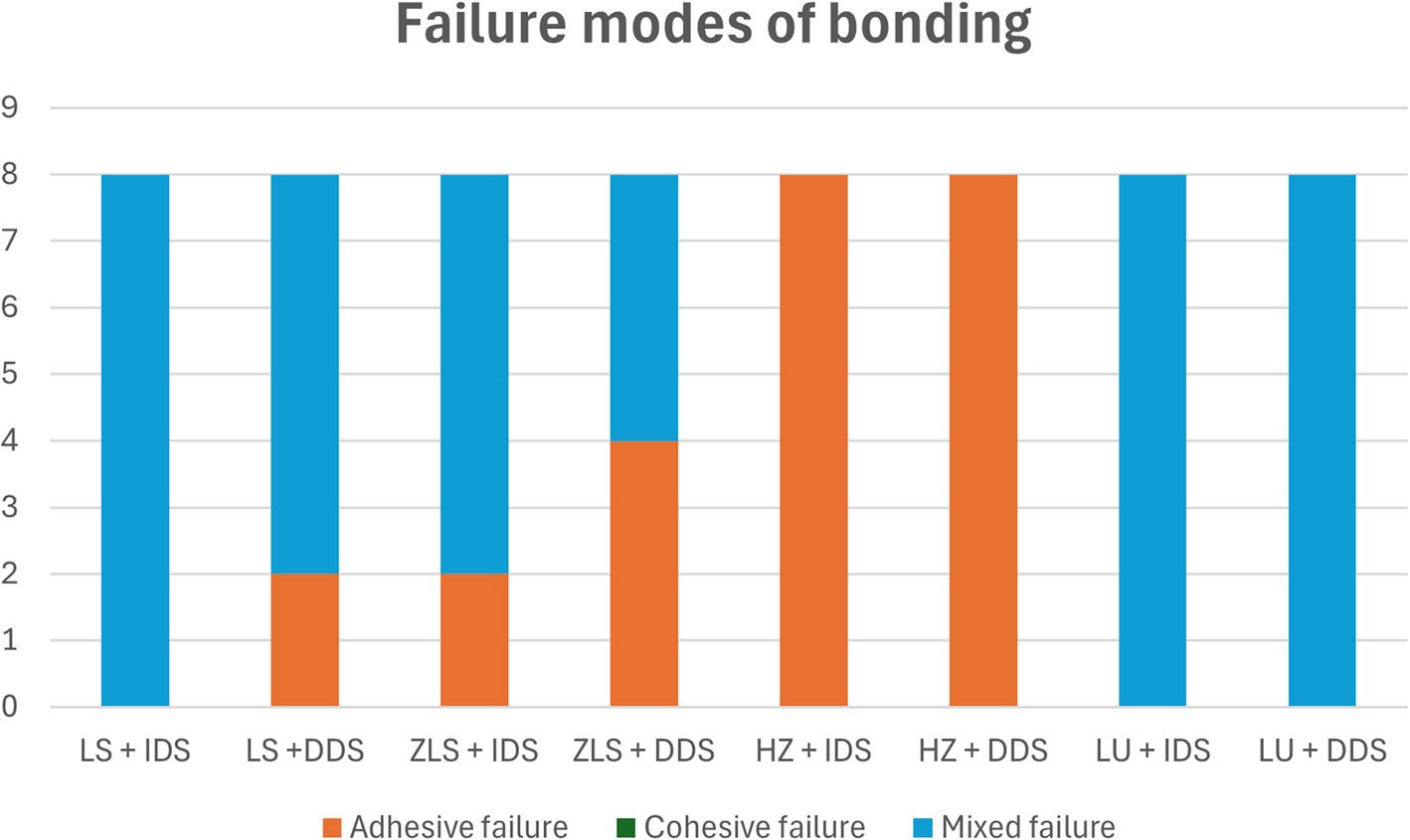
Analysis of failure modes of bonding

**Fig. 6:**
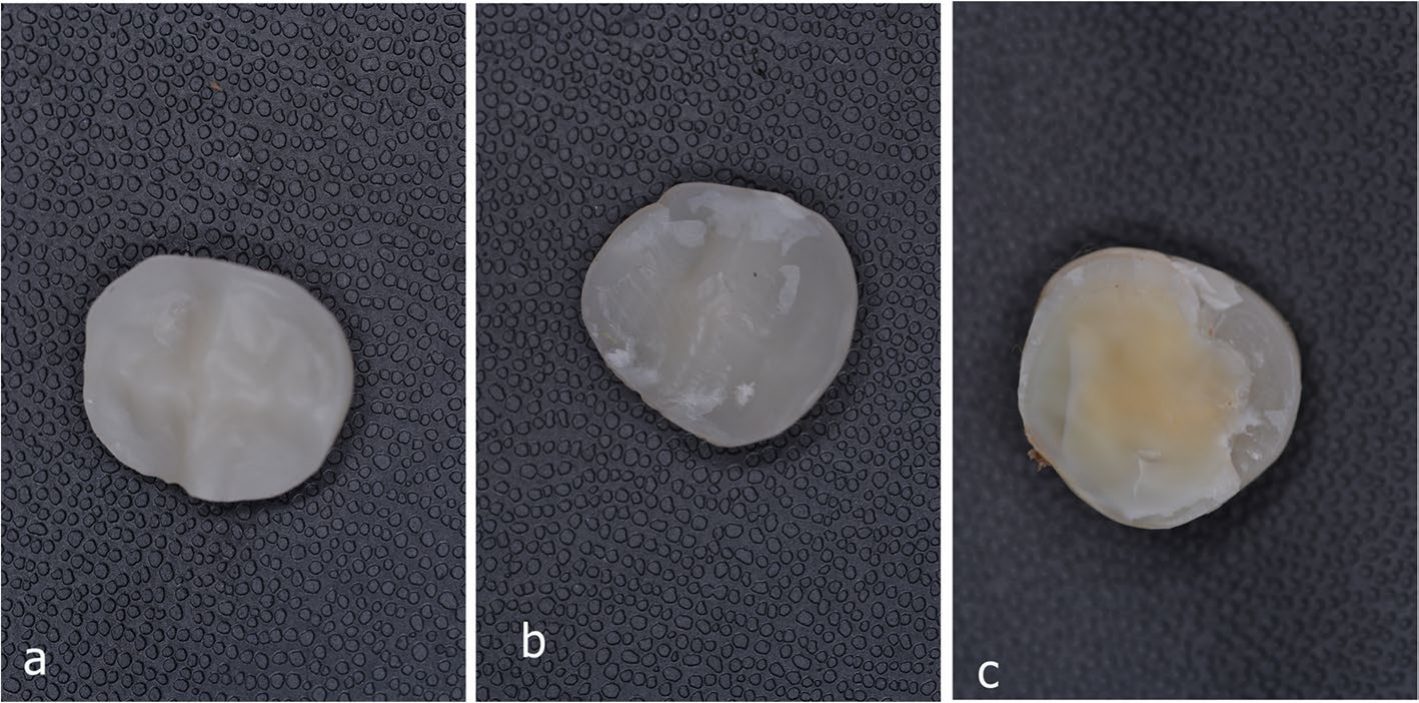
Representative images of failure modes after bond strength test, (**a**) adhesive failure; (**b**) mixed failure with small areas of resin remnants; (**c**) mixed failure with part of tooth bonded to occlusal veneer

The analysis of failure modes is presented in (Fig. 5). All LS + IDS veneers showed mixed failures while most of them with attached tooth parts, LS + DDS showed 6 restorations with mixed failures and 2 adhesive failures, this was similar to failures of ZLS + IDS where 6 veneers had (mixed failures with attached tooth parts) and the rest had adhesive failures, in ZLS + DDS group showed 4 mixed failures and 4 adhesive failures, this was in contrast to failures that occurred in HZ group (IDS and DDS) where all had adhesive failures, similarly LU veneers for (IDS and DDS) showed restorations of mixed failures with minute parts of attached resin cement to the fitting surface, (Fig. 6a-c). The cohesive failures were not presented in this study.

Subsequently, for fracture resistance the main effect of materials was investigated after eliminating the dentin sealing factor, the results are presented in (Table 6), there was significant increase in the fracture resistance of all studied materials than LU (*p* = 0.002^*^). HZ showed the highest fracture resistance (2225.8 ± 255.3N), followed by LS and ZLS (2210.3 ± 227.5, 2177.0 ± 278.2N, respectively), while the lowest was LU (1882.6 ± 321.7N).

**Table 6:** Comparison between the studied different materials according to Fracture resistance (N)

Fracture resistance (N)	Material				F (p)
	**LS** **(*n* = 16)**	**ZLS** **(*n* = 16)**	**HZ** **(*n* = 16)**	**LU** **(*n* = 16)**	
Min.	1807.7	1596.4	1718.2	1186.7	*F* = 5.651^*^ (*p* = 0.002^*^)
Max.	2641.5	2554.6	2576.6	2449.8	
Mean	2210.3^a^	2177.0^a^	2225.8^a^	1882.6^b^	
± SD.	227.5	278.2	255.3	321.7	
Median	2153.7	2222.4	2280.8	1977.3	

*SD* Standard deviation, *F* One way ANOVA test, Pairwise comparison between each 2 groups was done using Post Hoc Test (Tukey), *p p* value for comparing between the studied different subgroups

^*^Statistically significant at *p* ≤ 0.05. Means with Different letters ^(a−b)^ are significant

**Fig. 7:**
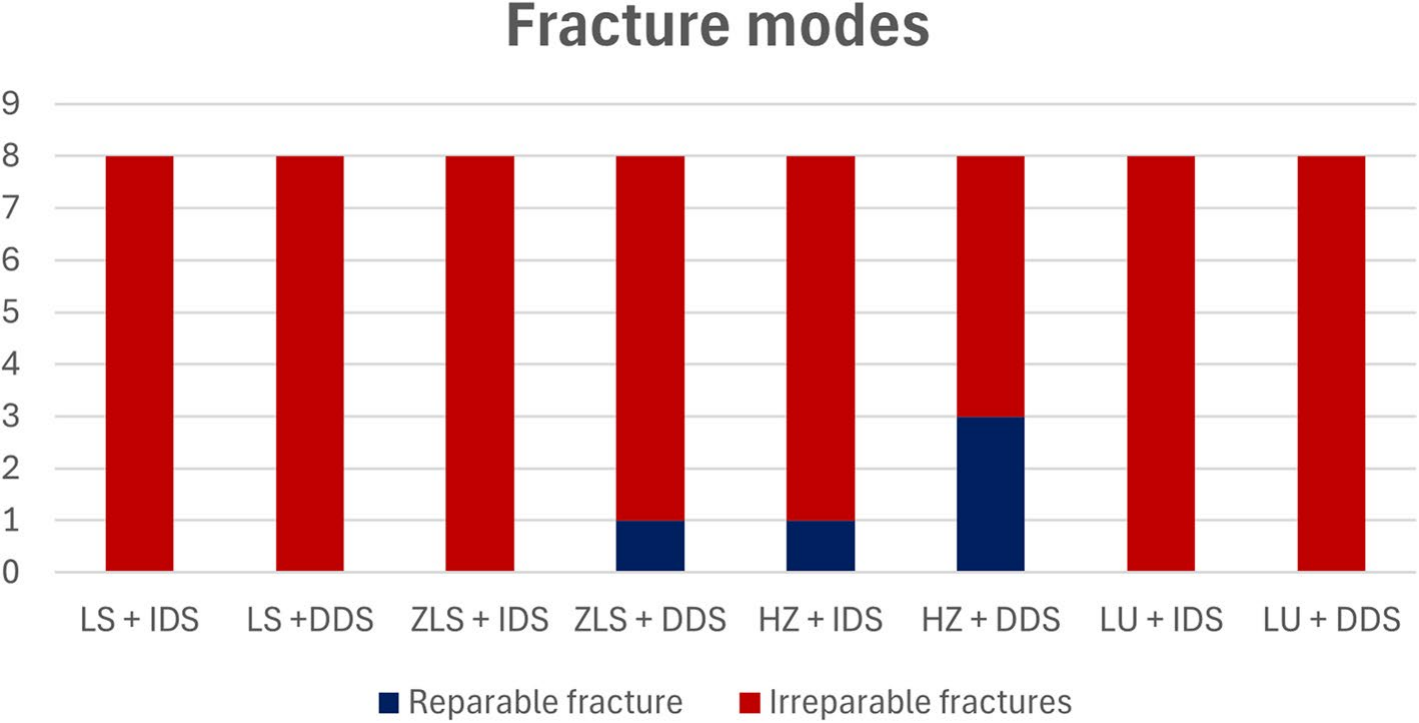
Analysis of fracture modes

**Fig. 8:**
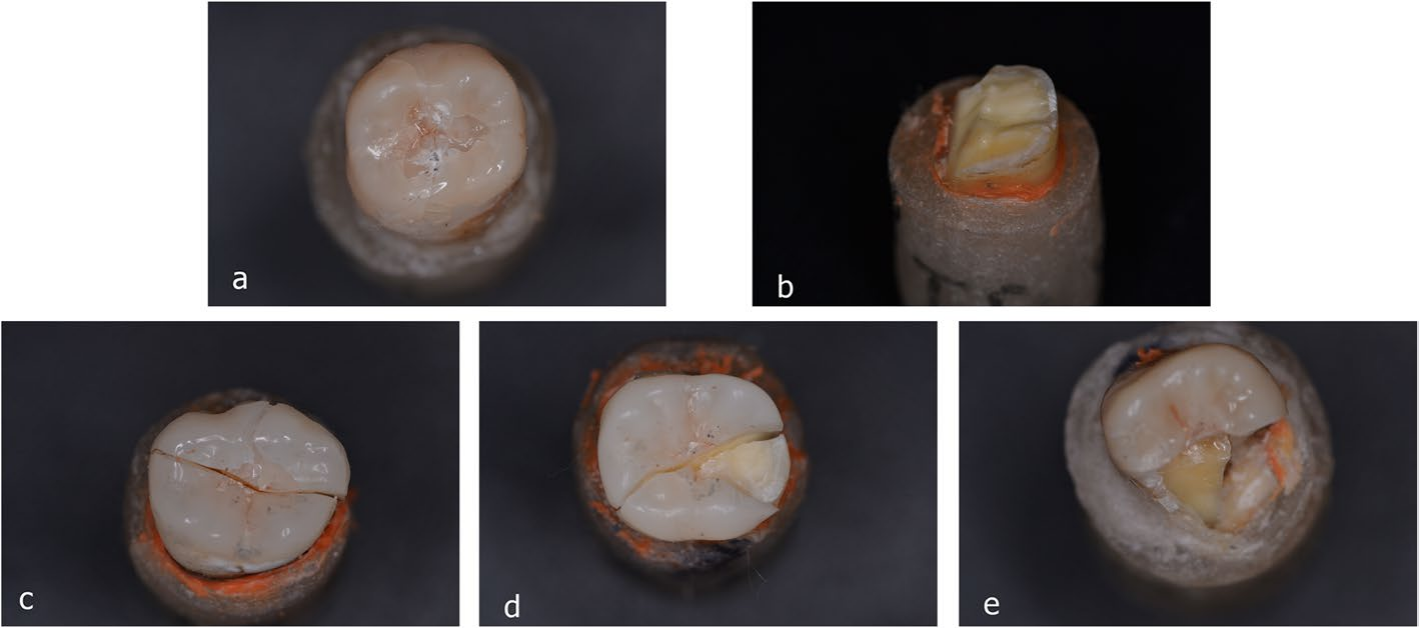
Representative images of fracture modes of failed specimens (**a**) reparable fracture showing crack formation within restoration without chipping; (**b**) reparable fracture tooth is indicated for more drastic restoration; (**c**, **d**, and **e**) examples of irreparable fracture with longitudinal fracture of the restoration and tooth

The examination of failure modes is presented in (Fig. 7), it showed a predominance of irreparable fractures for all specimens of all groups however the occurrence of reparable fractures were mostly in HZ group in DDS subgroup where the remaining part of the tooth can be prepared to receive another form of indirect restoration or root canal treatment followed by a post and core restoration, also it was noted that one veneer of ZLS + DDS showed in situ cracking of the veneer without fracture of restored tooth witch indicated re-restoration of tooth, (Fig. 8a).

**Fig. 9:**
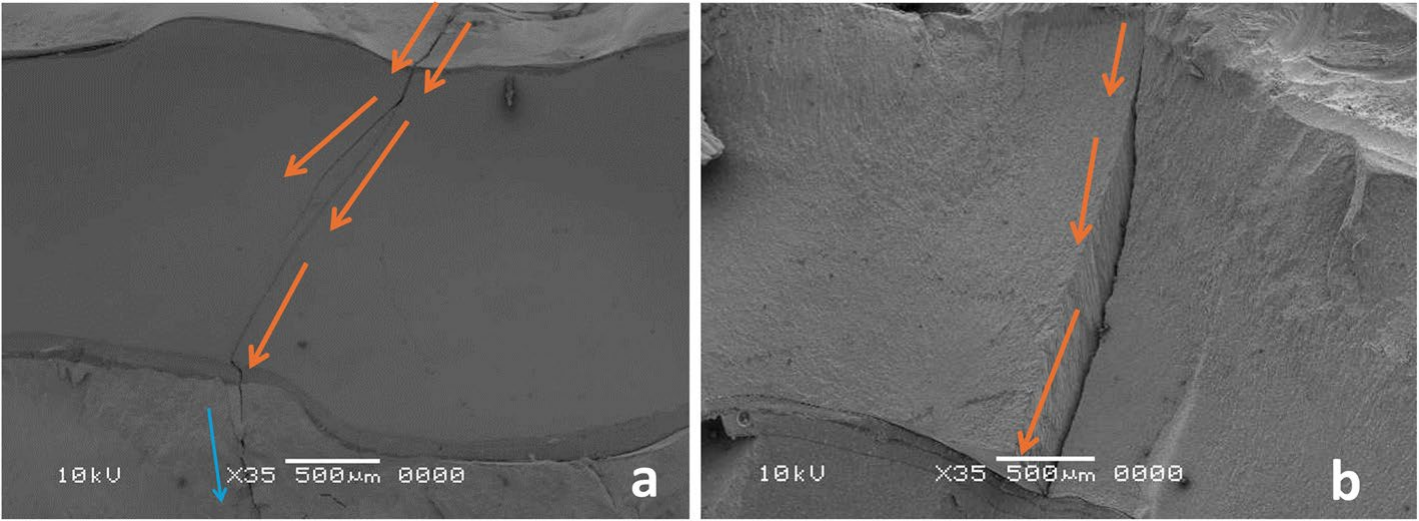
**a**, **b** Fractographic analysis of lithium disilicate restorations, (orange arrows) detect the propagated crack through the material and transmitted through the resin cement and tooth structure (blue arrow)

**Fig. 10:**
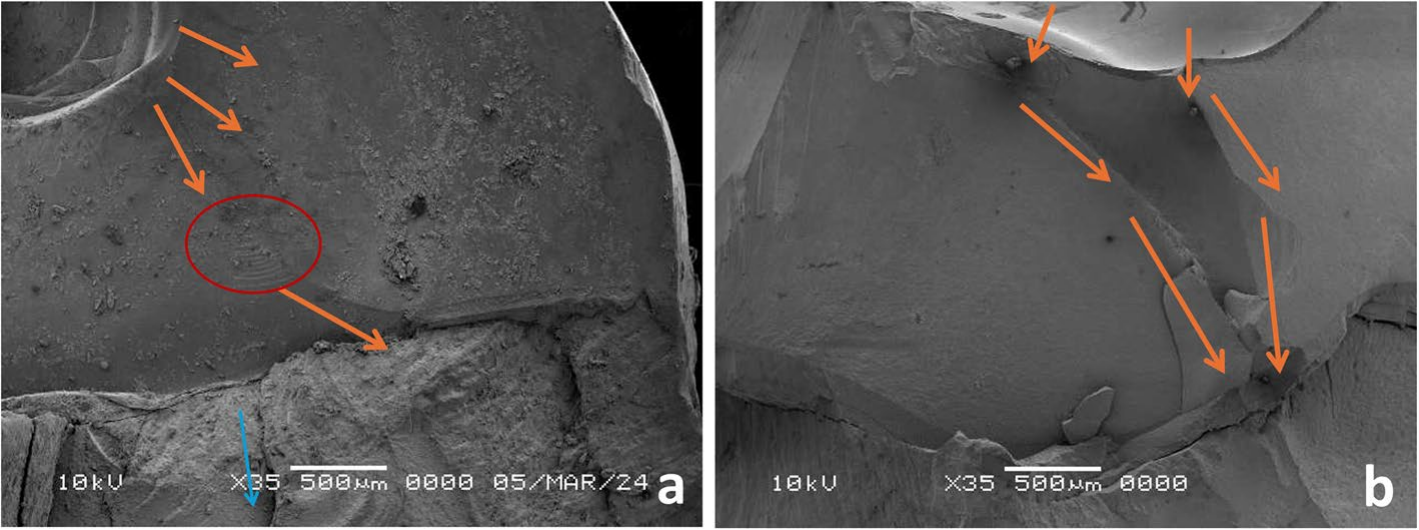
**a**, **b** Fractographic analysis of ZLS restorations, orange arrows detect the propagating cracks which showed arresting (red circle), twisting or redirecting their direction by the effect of impeded zirconia particles with less progress through tooth structure (blue arrow)

**Fig. 11:**
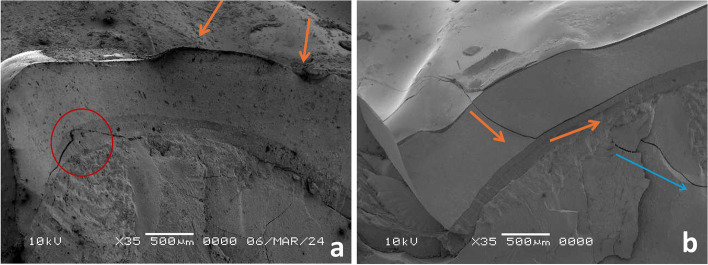
**a**, **b** Fractographic analysis of HZ restorations, the propagating cracks transformed (red circle) or extended from zirconia to the resin cement layer with a resultant debonding of the restoration, without direct communication of cracks through tooth structure (blue arrow)

**Fig. 12:**
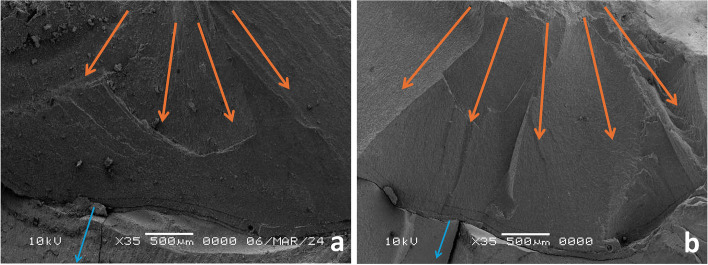
**a**, **b** Fractographic analysis of LU restorations, orange arrows detect the moving crack that proceeded in the form of radiating circular crack outward from the flaw, with further extension through tooth structure (blue arrows)

The fractographic analysis can disclose the fracture origin, and its direction, representative samples of each restorative material were presented, for LS the crack propagated through the material without any interferences for their progress and transmitted through the resin cement and tooth structure (Fig. 9a, b), while for ZLS the propagating cracks showed arresting, twisting or redirecting of their direction with less progress through tooth structure (Fig. 10a, b), for HZ the propagating cracks transformed or extended from zirconia to the resin cement layer with a resultant debonding of the restoration, without direct communication of cracks through tooth structure (Fig. 11a, b), and for LU the moving crack proceeded in the form of radiating circular crack outward from the flaw (Fig. 12a, b).

## Discussion

Based on the results of debonding forces and bond strength testing there was significant increase in both related to IDS application rather than DDS, also there was a significant effect of material selection on it, thus the first null hypothesis was rejected. Regarding the fracture resistance, the use of different dentin sealing strategies had no significant effect on it while the material selection affected it significantly hence the second null hypothesis was partially rejected.

The application of dental adhesives on exposed dentinal tissues directly following tooth preparation and before impression making in (IDS) protocol, led to creation and preservation of the hybrid layer during the temporization period or even before bonding of final indirect restorations especially for those prepared in the same dental visit [17]. Clinically IDS arrests the bacterial invasion and tooth sensitivity, moreover the thickness of dentin adhesive is already recorded before making the impression, so there will be no problem in restoration seating [18]. In contrary, in (DDS) protocol, the delayed application of dental adhesives directly before final bonding step and after provisional phase led to delayed formation of hybrid layer [17], and the indirect restoration is bonded to an almost contaminated or in other words not a freshly cut dentin [22]. In DDS the polymerization of dental adhesive before bonding step could interfere with the complete seating of the final restoration because of its thickness [16], so in this study the light curing of adhesive was done simultaneously with the final curing of resin cement to avoid the problem of incomplete seating of occlusal veneers.

Digital technology offered the ability to determine the surface area of prepared teeth, that could enable future researchers to precisely measure forces acting on teeth and restorations and to implement this clinically. In this study the measurement of actual surface area of occlusal preparation helped in the actual calculation of bond strength of occlusal veneer restorations. Las Casas et al., [39], found that the acting debonding forces on restorations can be clinically calculated using a sensor film between occluding teeth through measuring the tangential forces, generally the force magnitude varied differently by time during occlusion cycles without a definite pattern and differed according to different types of food and their consistencies. The tangential components of forces responsible for creation of debonding forces are nearly constant during occlusion and ranges between 30N and up to maximal peak of 140N [39].

For calculation of bond strength in this study, the applied debonding forces were automatically recorded during the test, and it was found that all occlusal veneers were de-bonded at higher forces than the clinically applied forces in patient’s mouth, as discussed earlier. Application of IDS increased the bond strength, however the results of bond strength calculation were lower than those recommended by literature, where it should be at least 17–20 MPa to dentin to be accepted clinically [40]. In this study, bonding of the occlusal veneers to occlusal surfaces could lead to variations in the surface area of prepared teeth, however, the adequate teeth selection and preparation resulted in obtaining surface areas of all teeth with no significant variations in different groups (*p* = 0.067), and the surface area ranged from (95.7834 to 111.3634 mm^2^). This means that debonding forces were divided by wide surface areas of the occlusal table of restored teeth, therefore, the bond strength results were decreased due to the increased surface area [41–43], whereas the values of debonding forces were higher than the clinically applied forces [39].

In literature it was found that adhesive bonding of indirect ceramic restorations to teeth improved their retention, fracture resistance, and subsequently the survival rate [35]. The results of this study were in accordance to results of previous studies which found that IDS process significantly increased the dentin bond strength and the longevity of indirect restorations [16, 17, 19–21]. Also, application of IDS improved the bond strength of occlusal overlays made of zirconia reinforced lithium disilicate and bonded to pre-measured flat surfaces of exposed dentin of mandibular molars with no statistically detrimental effect of IDS on the bond strength of etched glass ceramic to dentin, Reboul et al., [26]. Meanwhile, it was found that using 3- step etch and rinse adhesives is more useful than simplified adhesives, as recommended by Dalby et al., [1]. On the contrary, DDS resulted in higher bond strengths than IDS bond strengths using a 2-step self-etch dentin adhesive, Falkensammer et al., [44].

Nowadays, the universal adhesives are preferable to be used because they offered great benefits through their chemical bond to dental substrate and different restorative materials in addition to the original micromechanical bonding to tooth structure [45]. Universal adhesives are also called multimode adhesive, as it can be used either as total etch, self-etch or selective enamel etch modes, in this study selective enamel etching was used for improvement of the bond strength and marginal sealing to enamel, while on dentin surfaces, the self-etch mode was selected not to jeopardize the bond strength or affect the tooth sensitivity clinically in patients [37, 45]. All bond universal adhesive is ultra-mild with PH 3.2, that would cause mild etching of enamel if used in self-etch mode without an additional enamel etching, while regarding dentin, the self-etch mode was selected to keep its inorganic content for obtaining durable adhesion by chemical bonding of universal adhesive, which could be responsible for increasing the bond strength as obtained by Flury et al., [32]. Similarly, selective etching of enamel enhanced the bond strength of self-etch adhesives to levels comparable to those obtained with traditional total etch adhesives, Erickson et al., 2009 [33], thus selective enamel etching was important in obtaining the results of this study.

In IDS protocol, the application of adhesive and precuring of it retained the formed hybrid layer and prevent collapse of the collagen network during final bonding of restoration by the pressure exerted during seating of the restoration and the resin cement [16, 46]. This could be responsible for increasing the adhesive bond strength with better distribution of stresses throughout the formed hybrid layer [16]. In agreement with this, bond strength was improved in IDS by Clearfil SE Bond in comparison to DDS, and they related this to the amount and size of fillers present in the dental adhesives where the smaller the filler size allowed for their deeper penetration through collagen fibrils of clean uncontaminated dentin surface directly after preparation, Choi et al., [20].

For bonding of LS and ZLS veneers, application of Hydrofluoric acids (HF) was performed for dissolving of glass matrix and exposure of crystal structure [34], the resulted etched surfaces had 3D porous structure through exposure of randomly oriented and densely distributed numerous elongated fine-grained lithium disilicate crystals for LS and bean-like lithium silicate crystals for ZLS, that increased the surface roughness and bonding area for better wettability of the ceramic surface by the applied ceramic primer and resin cement [34, 47], whereas ZLS showed smaller crystal phase compared to LS [48], this could be related to the lower bond strength obtained results of ZLS in comparison to LS.

Regarding the monolithic zirconia restorations, the increase in bond strength was related to better bond strength for MDP-containing luting agents accompanied with air borne particle abrasion as surface treatment of zirconia [49]. It was reported that the application of priming agents (Z-prime plus) containing hydrophobic (MDP) phosphate monomer and carboxylic acid monomer resulted in high initial and post-thermocycling bond strengths [30]. In a study by Rigos et al. 2019 [49], IDS significantly improved the bond strength of monolithic zirconia to dentin by using MDP-containing luting agents, and the adhesive failure was the least to occur, this could be related to absence of thermocycling in the methodology, also the bonding of zirconia discs directly without temporization period and finally the used surface treatment of zirconia itself could be responsible for the bonding improvement.

While concerning Lava Ultimate restorations, they are resistant to HF acid etching due to the existing zirconia nanomer fillers in their composition [50]. They were sandblasted by 50 µm Al_2_O_3_ for roughening of fitting surface, that resulted in increased surface area for bonding, but it was found that, the created sharp irregularities of fitting surface could decrease surface wettability by resin cement, also they caused microcracks in the ceramic surface, which could be responsible for their premature failures [50]. In contrast to this, higher bond strength to LU restorations was obtained by using 110 µm particle size for sandblasting especially when accompanied with silane application, although silane application could be insignificant because of its preexisting polymer content [51], in this study ceramic primer was used for surface pretreatment of LU veneers according to manufacturer’s instructions.

A self-adhesive dual cure resin cement (Duo-Link universal cement system) was used in this study [30], it has advantage of showing satisfactory mechanical properties, and are indicated to cement restorations that could obstruct enough light energy from being transmitted to the cement [52]. The increase in filler content of this cement, reflected a better bond strength, where mixed type failures were most common failure type, since they were not only at the interface, but also involved parts of bonded tooth structure [52], while regarding the HZ restorations, adhesive failure predominate with apparently clean fitting surface of veneers that could represent difficulty in bonding of zirconia restorations even though the enormous trials for improvement of its bonding [53]. The higher number of adhesive failures could be related to the higher flexural strength of HZ with resultant stress concentration at the boundary of rigid (HZ) and flexible (resin cement) during loading with a resultant failure at this interface [50].

In the literature, it was noted that ceramic restorations adhesively bonded to dentin have lower fracture resistance than those bonded to enamel, and the use of IDS bonding protocol improved both the adhesion and fracture strength of veneers, while most of bonding failures occurs at dentin-cement interface as it was considered the weakest link for adhesive restorations, also the smaller the areas of exposed dentin the less benefit from IDS technique, Gresnigt et al., 2016 [46]. Indirect restorations bonded by the adhesive resin cements offered superior fracture resistance due to better reinforcement of tooth structure and ability to form a single unit between prepared tooth and occlusal veneer that allowed for dissipation of forces to the tooth structure, periodontal ligament and alveolar bone [38]. This resulted in improvement of fracture resistance, and significant increase in fracture resistance of occlusal veneers made of polymer infiltrated ceramics and bonded by IDS in comparison to DDS, Teche et al., [24], and this was related to the thickness of adhesive which supported the brittle occlusal veneers, as the adhesive was placed two times; one immediately after preparation and once more during the bonding procedure, with a resultant increase of its film thickness.

In this study, it was found that application of IDS had no significant increase in the fracture resistance of occlusal veneers made from different ceramic materials, while this was significantly correlated to the material type only. The highest fracture resistance was recorded by HZ followed by LS and ZLS restorations without any significance between them, while the lowest fracture resistance was recorded by LU with highly significance from the other used materials, meanwhile, all results are higher than clinically applied forces. Obviously, the fracture resistance of the occlusal veneers interrelated mostly to their mechanical properties, this in accordance with the findings in the literature, where the fabricated veneers from high flexural strength materials, had greater fracture resistance [47].

New high translucent zirconia generations with more yttria content do not undergo transformation toughening, so they have lower mechanical properties [53] but still they have higher fracture load than silicate glass–ceramics [54]. In this study the results of fracture resistance were not correlated to the significant improvement of the bond strength resulted from application of IDS, meanwhile for the fractographic analysis it was noted that the forces were transmitted from the zirconia with higher modulus of elasticity to the cement with lower elastic modulus, so stresses concentration occurred at the internal surface of restoration [50, 55, 56], accompanied with subsequent debonding between zirconia and resin cement, that could highlighted the cause of lower bonding potential of HZ restorations. This was in accordance to another study by Zamzam et al.,2021 [4], who analysed the failure of high translucent zirconia, hybrid ceramic, and lithium disilicate occlusal veneers under lateral static loading, they found that zirconia veneers showed the highest resistance to failure, with interfacial debonding was mainly presented by zirconia while the other two material failed mainly by veneer fracture at lower loads.

Concerning the high fracture resistance of LS restorations, it could be related to the material’s high mechanical strength as related to its interlocked microstructure of the needles and flakes shape of lithium disilicate crystals where the crack is initiated in silica matrix and further propagation was hindered by lithium disilicate crystals, causing deflection, branching of cracks [13, 57], this effect was noticed in the fractographic analysis, where the high fracture energy leads to fasten the crack propagation, with subsequent more bifurcation if it [58].

Regarding the LS CAD material it was noted the highest flexural strength was related to medium translucency blocks, followed by low translucency blocks and the lowest is the high translucency [57], the selected LS CAD/ CAM blocks in this study were of medium translucency because translucency is of no importance in restorations of posterior region and this could be the cause of high fracture resistance of LS in this study.

While for ZLS restorations it was thought that inclusion of zirconia would increase its fracture resistance, they showed high results with no significant differences with the other materials except for LU, ZLS showed close similarity to structure of LS, but the crystal reinforcement by zirconia particles increased its toughness as in tetragonal zirconia (t-ZrO_2_) restorations, it was noted in the fractographic analysis that the inclusion of zirconia particles were beneficiary for arresting the cracks and redirecting of it, with lesser progression through the tooth structure [59], nevertheless zirconia particles acted as nucleation sites and hindered the crystal growth resulting in smaller size of crystals, and this could be the cause for lower fracture resistance of ZLS than LS [57, 60].

The high fracture resistance of LS occlusal veneers agreed with Krummel et al., [29], who related it to the high bond strength by selective enamel etching and self-etch adhesives, meanwhile, bonding to dentin with lower elastic modulus resulted in dissipation of applied forces and improvement of fracture resistance of veneers than those bonded to enamel.

Considering the stiffness or rigidity of the restorative materials, materials with higher elastic modulus are vulnerable to greater stress concentration inside themselves or at the internal surface of it, and less stresses are delivered to the underlying tooth structure whereas decreasing the elastic modulus of the materials means lower stress concentration in the material and greater stresses are delivered to underlying tooth structure [13, 56, 61]. Therefore, the Lava Ultimate hybrid ceramics have the shock absorbing features; a propagating crack is dispersed as it passes through its resin component, due to its low elastic modulus, the crack velocity was less than that of glass ceramics, so the moving cracks were nucleated, and circular crack radiates outward from the flaw [58].

Lava Ultimate restorations showed high fracture resistance values, but they were significantly lower than all other types (LS, ZLS, and HZ). This could be related to air abrasion that was required for surface treatment before bonding, as it could lead to negative effect on flexural strength, and weakening of the restorations, Muhammed et al., [62]. On the other hand, it was thought that the polymeric materials with its resinous matrix would have superior bond strength when compared to ceramic materials, with resultant higher fracture resistance values [63], but this was not parallel to findings of this study.

Other studies confirmed results of this study [12, 38, 64] with greater fracture resistance for LS ceramic restorations than those fabricated from hybrid ceramics. Additionally, other studies found that zirconium oxide ceramic restorations had higher fracture resistance than lithium disilicate and hybrid ceramic restorations [4, 14, 63]. Whereas, occlusal veneers made from different materials (CAD/CAM lithium disilicate, ultra-translucent zirconia and polymer infiltrated ceramic materials) showed no significant differences [30], this was related to ability of tooth-restoration complex to withstand final load potential other than the mechanics of restorative materials themselves, and strong adhesive bonding is able to strengthen weak ceramics and balance forces applied to it, [12, 30], especially with universal adhesive application and reinforcement of fracture strength through efficient bonding to tooth structure.

Most of the occurring fracture modes in this study were irreparable with longitudinal fracture of the tooth that exposed the base of the pulp chamber, this could be related to the external peripheral enamel bevel, which redirected all eccentric forces to the long axis of the tooth [65]. Lava ultimate veneers was thought to have reparable fractures due to its low modulus of elasticity however they resulted in irreparable fractures, this was in accordance with a study which found that Lava Ultimate at 1.5 mm thickness accumulated the applied stresses on its surface when exceeded the proportionality level and dissipated them along tooth restoration interface with increased liability to catastrophic failures [38]. Similarly, stresses occurring in the cement layer were higher in hybrid ceramics followed by zirconia and lithium disilicate while stresses accumulated in occlusal veneers themselves were higher for zirconia thus, higher liability of reparable fractures occurring mainly in HZ [4, 56].

In this study it was revealed that fracture resistance of occlusal veneers fabricated from different materials and bonded with either (IDS or DDS) surpassed the maximum chewing forces in the mouth with a maximal biting force of 800N in molar region [66], whereas in patients with parafunctional habits forces, they varied from 780 to 1120 N in molar region [67]. The correlation between the normally achieved fracture resistance values and load-bearing capacities of the occlusal veneers in this study, could be correlated to mechanical properties of restorative material themselves, and to the bonding properties of the resin cement to exposed dentin as proper adhesion commands the longevity of the restoration [14].

The limitations of this study include the difficulty to mimic the biomechanics of the oral cavity, absence of dentinal fluids with its effect on adhesive bonding and changes of pH values. These factors are critical in simulation of the clinical situation and should be considered in future in-vitro studies. Moreover, the use of immediate dentin sealing without temporary restoration was not examined in this study to simulate the clinical condition of unavailability of intra oral scanner in the dental clinic, however, the effect of present remnants of temporary material should be taken into consideration, where better bond strength values could be obtained.

Further investigations should be done on clinical levels to accurately document the survival and encounter the failures and complications of occlusal veneers, moreover, bond strength test can be performed following the direction of clinically applied force in the oral cavity, as it should be at an angle to the buccal cusp instead of being parallel to the tooth restoration interface.

## Conclusions

Within the limitations of this in-vitro study, it can be concluded that.

1. Immediate dentin sealing had a positive impact on the required debonding forces of occlusal veneers made of different materials and their bond strength without significant effect on their fracture resistance.
2. Material selection is critical for obtaining adequate restoration performance; however, all of the used materials can withstand lateral and occlusal forces higher than the values recommended for restoring posterior teeth.
3. Fractographic analysis of fractured specimens emphasized the importance of understanding the structure and mechanical properties of used materials to secure better prognosis of ceramic restorations in different clinical conditions.
4. The use of HZ for occlusal veneer restorations is highly recommended due to its high fracture resistance and protection of underlying tooth structure from crack propagations, however it was not the best regarding the bonding performance with no significance from LS and ZLS, where both were also satisfactory for use due to their adequate bonding performance and fracture resistance values.

## Data Availability

Data is provided within the manuscript and supplementary information files are available on request.
